# Pediatric upper lip myopericytoma: a case report and comprehensive review

**DOI:** 10.1186/s12903-024-04106-y

**Published:** 2024-04-20

**Authors:** Bin Wei, Gui Liu, Kun Li, Hongzhi Quan

**Affiliations:** 1 Department of Oral and Maxillofacial Surgery, Xiangya Stomatological Hospital & School of Stomatology, Central South University, Changsha, 410008 Hunan P.R. China; 2 Hunan Key Laboratory of Oral Health Research & Hunan 3D Printing Engineering Research Center of Oral Care & Hunan Clinical Research Center of Oral Major Diseases and Oral Health & Xiangya Stomatological Hospital, Xiangya School of Stomatology, Central South University, Changsha, 410008 Hunan P.R. China; 3 Department of Oral Pathology, Xiangya Stomatological Hospital & School of Stomatology, Central South University, Changsha, 410008 Hunan P.R. China

**Keywords:** Myopericytoma, Pediatric oral diseases, Pathology, Immunohistochemical staining, Hematoxylin and Eosin staining

## Abstract

**Background:**

Myopericytoma is a rare spindle cell tumor of mesenchymal origin, typically benign, characterized by concentric proliferation of tumor cells around blood vessels within subcutaneous tissue. It primarily occurs in middle-aged adults and is often located in distal extremities, although cases have been reported in proximal extremities and head-neck regions. However, occurrences within the oral cavity are exceedingly rare. To date, literature reviews have identified only two cases in children under 10 years old and reported only five cases of myopericytoma occurring in the lip region. We provide a comprehensive review and analysis of all documented cases to better understand this condition.

**Case presentation:**

A 7-year-old girl presented to oral and maxillofacial surgery with the discovery of a painless mass on the inner aspect of the upper lip. The diagnosis of myopericytoma was confirmed by histological examination (HE staining), alcian blue staining, and immunohistochemistry.

**Conclusions:**

Following surgical excision, there were no signs of recurrence at a 3-month follow-up. The pathological diagnosis of myopericytoma is quite challenging, and immunohistochemical testing is necessary.

**Supplementary Information:**

The online version contains supplementary material available at 10.1186/s12903-024-04106-y.

## Background

Myopericytoma (MPC) is a histopathologically benign subcutaneous tumor distinguished by the concentric organization of oval to spindle-shaped myoid cells surrounding delicate vascular channels, exhibiting a distinctive concentric perivascular cell proliferation [1]. Myopericytoma can manifest at any age but is most prevalent in middle-aged individuals. It typically originates in subcutaneous tissues, frequently impacting the distal extremities, but it can also arise in the proximal extremities, neck, thoracic spine, and various other regions. Nevertheless, documented cases of MPC within the oral cavity are exceedingly scarce [2]. Clinically, it manifests as painless subcutaneous nodules characterized by slow growth, and the disease course may span several years. While it frequently appears as a solitary nodule, multiple lesions are not uncommon. Multiple lesions often develop asynchronously, typically affecting a specific anatomical region, such as the foot or head-neck region [3]. To date, a comprehensive literature review has unveiled a total of 41 documented cases of myopericytoma manifesting in the oral cavity, summarized in Table 1. Cases classified by gender, age, and anatomical site are summarized in Supplementary Table 1. In this report, we present a case involving myopericytoma located in the upper lip of a 7-year-old girl.

**Table 1:** The Case Reports of Myopericytoma Occurring in the Oral Cavity

No	Year-author	Age/ gender	Location	Follow up	Immunohistochemistry				
					α-SMA	Desmin	CD34	Caldesmon	Ki-67
1	2005-LI [48]	48/F	Right submandibular	No recurrence 8 months	+		-		
2	2007-Ide [24]	45/F	Right buccal mucosa	No recurrence 9 years later	+	-	-	+	
3	2007-Datta [11]	36/F	on the anterior left lateral tongue	No recurrence	+		-		
4	2008-Maheshwari [44]	42/M	Right neck	NA	+	-			
5	2008-Laga [27]	72/M	on the edentulous alveolar ridge of the right mandible	No recurrence 18 months	+	-	-	+	
6	2009-Chu [17]	41/F	right parotid gland	No recurrence 9 months	+	-	-		
7	2009-Sapelli [21]	28/M	Upper lip	No recurrence 3 years	+	-			
8	2010-Terada [46]	56/F	Neck	No recurrence 4 years	+	-	+	+	8%
9	2010-Kuczkowski [16]	65/M	Left parotid gland	No recurrence 2 years	+		-		
10	2010-Xia [4]	43/F	right parotid gland	Recurrence twice	+	-	-		
11	2011-Rho [45]	70/F	left side of the neck	No recurrence 20 months	+		+		
12	2012-Jung [3]	40/F	Left parotid gland	No recurrence 4 years	+	-	+		
13	2012-Terada [28]	61/M	right cheek mucosa	No recurrence 6 months	+	-	-		40%
14	2013-Wu [15]	42/F	right parotid gland	No recurrence 5 years	+	+	-		
15	2013-Kim [49]	44/F	Right cheek	NA	+	-	-	+	
16	2013-Akbulut [9]	61/F	left mid-lateral tongue	No recurrence 18 months	+	-	-		5%
17	2014-Bates [14]	66/M	right parotid region	No recurrence 18 months	+			+	
18	2015-Mathew [47]	12/M	coronoid process	No recurrence 2 years	+	-	-	-	
19	2015-Vasenwala [20]	14/M	Upper lip	No recurrence	+		+Weakly		
20	2016-Chaskes [51]	23/F	left supraclavicular fossa	No recurrence 5 months	+	-	-		
21	2016-Prado–Calleros [52]	38/F	right carotid triangle	No recurrence 8 years			+		
22	2017-Rubino [8]	48/M	Tongue base	NA				+	< 5%
23	2018-Strayer [7]	42/F	lower lip on the left side	Recurrence 3 months	+		-		
24	2019-Ju [6]	46/F	Parotid	No recurrence 56 months	+	- or + focally	- or + focally	-	< 8%
25		10/M	Tongue	No recurrence 51 months					
26		41/F	Buccal	No recurrence 27 months					
27		61/M	Tongue	No recurrence 25 months					
28		62/F	Submandibular	No recurrence 8 months					
29	2019-Ralli [19]	46/M	upper lip	No recurrence 8 months	+	-	+		< 5%
30	2020-Cebeci [53]	44/F	forehead	8 months	+	-	+focally		
31	2020-almeida [26]	12/F	gingival on the palatine surface	No recurrence 5 years	+	-			35%
32	2021- Porat Ben Amy [25]	6/M	posterior right maxilla	No recurrence 8 years	+				
33	2021-Sawaf [42]	56/F	Left mastoid	No recurrence 6 months	+		-		
34	2021-Pan [13]	62/M	left parotid gland	No recurrence 17 months	+		-		
35		48/F	right parotid gland	No recurrence 5 years	+	-	-		
36	2022-Tan [18]	24/F	Lower lip	No recurrence 43 months					
37	2022-Roig [12]	30/F	parotid gland	No recurrence 5 months	+	-	-	+	15%
38	2023-Slack [54]	21month-old/M	perioral region	No recurrence 3 months	+	-	+		

a. The following cases are not listed in the table: one occurred in a 42-year-old male on the tongue [10] and one occurred in a 22-year-old male in the temporal region [55] due to lack of treatment methods and follow-up information; one occurred in an 80-year-old female in the left neck area [29], alive with disease 24 months after marginal excision and with liver metastases at 14 months

b. All other patients listed in the table underwent complete surgical excision

## Case presentation

**Fig. 1:**
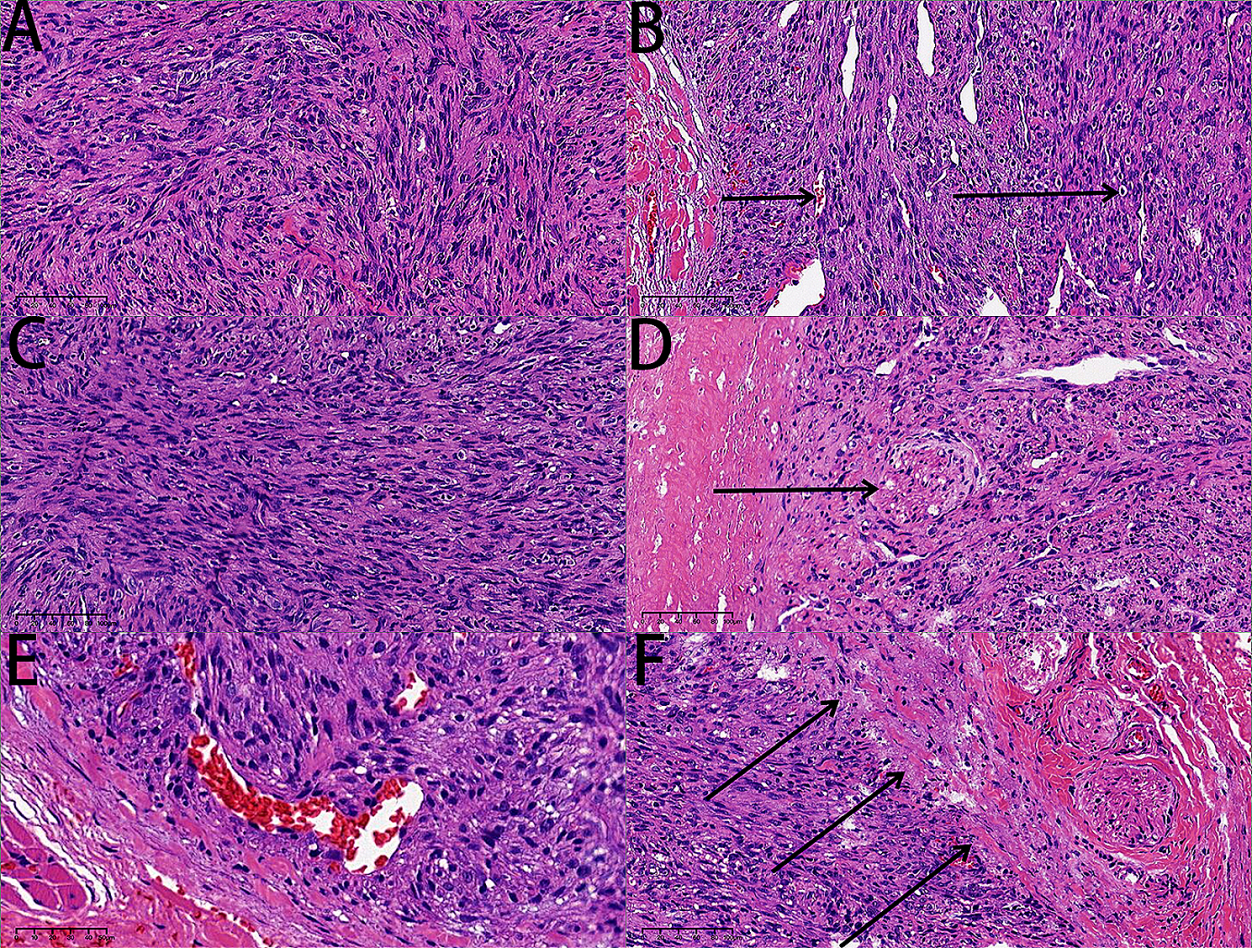
(**A**) relatively uniform oval or spindle-shaped myoid cells arranged in a whirlpool pattern (box) (bar = 100 μm). (**B**) In the clefts, a few red blood cells (short arrow) were observed within the blood vessels, and some tumor cells exhibited vacuolar changes with nuclei located centrally within the cells (long arrow), which is typical feature of myopericytoma. (bar = 100 μm). (**C**) The myoid cells exhibited acidophilic cytoplasm, uniform nuclear chromatin, and lacked notable nuclear pleomorphism (bar = 100 μm). (**D**) Nerve fiber (arrow) could be observed in cross-sections located within the center of the tumor (bar = 100 μm). (**E**) The presence of plump myoid cells encircling rounded, thin-walled vessels (bar = 50 μm). (**F**) The fibrous capsule had a clear boundary (arrows) with the surrounding normal tissue (bar = 100 μm)

**Fig. 2:**
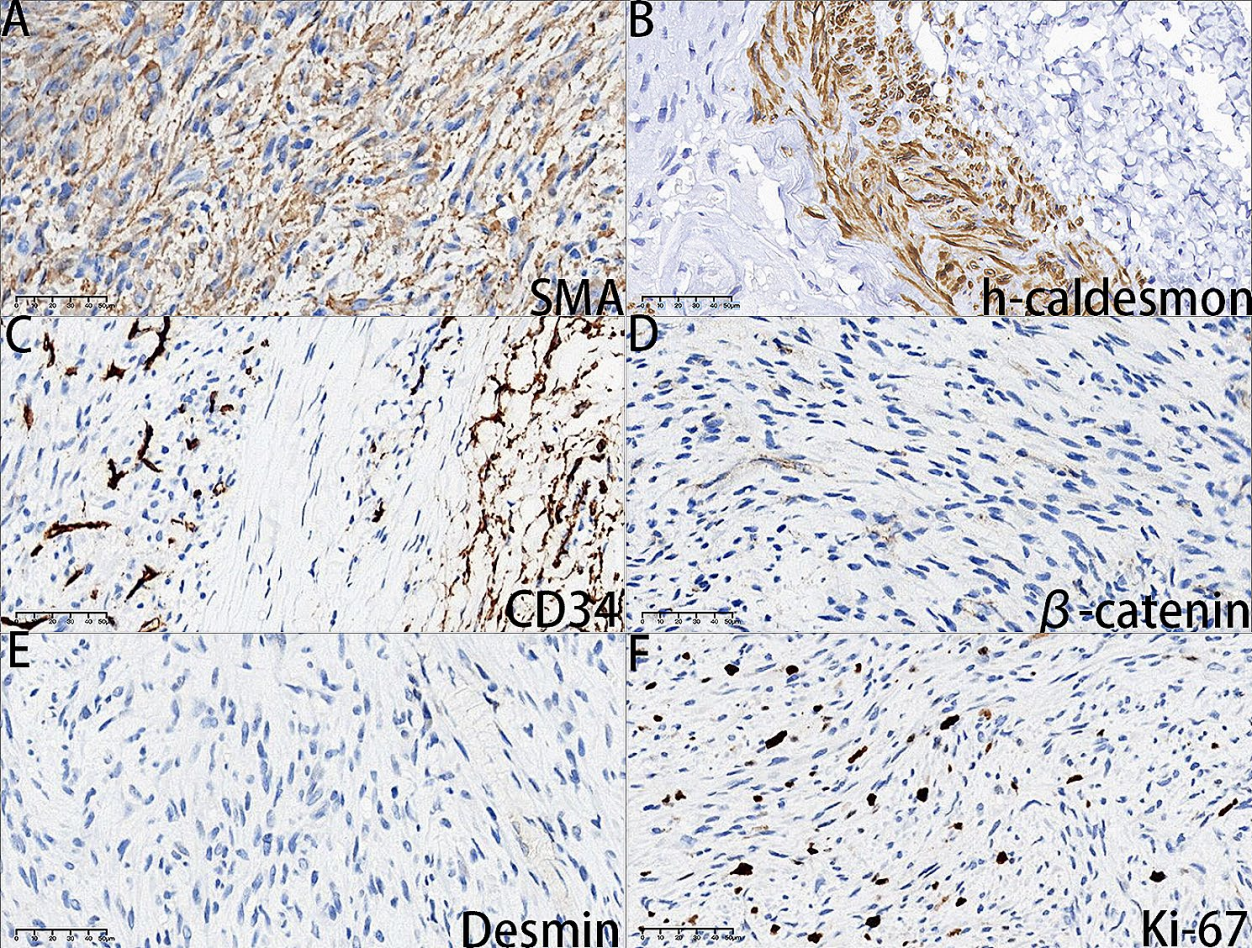
(**A**) Positivity for α-SMA accentuates the perivascular arrangement (bar = 50 μm). (**B**) The expression of h-caldesmon is focal to smooth muscle in visceral organs and blood vessels, as well as myoepithelial cells. (bar = 50 μm). (**C**) The tumor cells are negative for CD34 but endothelial cells lining the lumen of small blood vessels are positive. (bar = 50 μm). (**D**) Positive results for β-catenin can serve as evidence to exclude solitary fibrous tumors. (bar = 50 μm) (**E**) The tumor cells are negative for Desmin (bar = 50 μm). (**F**) The tumor cells showing a high Ki-67 labeling index (20%). (bar = 50 μm)

On August 10, 2023, a 7-year-old girl was brought to Xiang Ya Stomatological Hospital in Hunan Province, China. Her mother reported the discovery of a painless swelling on the inner aspect of the upper lip two weeks before the visit. The patient had a history of overall good health, with no known drug allergies or other systemic medical conditions. During a specialized examination, an upper lip lesion was identified on the left side. The lesion exhibited a color resembling that of the surrounding mucosa, had a firm texture, unclear borders, and dimensions measuring approximately 0.5 × 1.0 × 1.0 cm. The treatment involves surgical excision. Subsequently, the excised lesion was dispatched to the pathology department for thorough examination. Following consultation with the pathology department at Xiang Ya Hospital, the comprehensive pathological assessment, which encompassed hematoxylin and eosin (H&E), alcian blue staining and immunohistochemistry, conclusively established the diagnosis of a benign myopericytoma originating from the mesenchyme in the upper lip. Histological Features: Grossly, the lesion displayed indistinct boundaries with surrounding tissues and was enclosed by a complete capsule, measuring approximately 0.5 × 1.0 × 1.0 cm. Microscopic examination of hematoxylin & eosin and alcian blue stained sections revealed the following characteristics: First, the presence of relatively uniform oval or spindle-shaped myoid cells arranged in whirlpool patterns. Additionally, there were some blood vessels with occasional red blood cells in the lumens, and certain tumor cells displayed vacuolar changes with centrally located nuclei, which is typical feature of myopericytoma. And the myoid cells exhibited acidophilic cytoplasm, uniform nuclear chromatin, and lacked notable nuclear pleomorphism. Furthermore, mucinous degeneration was detected within the matrix between whirlpool structures by alcian blue staining. Moreover, the fibrous capsule exhibited distinct demarcation from the surrounding normal tissues. Cross-sections within the central part of the tumor revealed the presence of nerve fiber. Equally important is the presence of plump myoid cells encircling rounded, thin-walled vessels. (Fig. 1A∼1F) and (Supplementary material Fig. 1). To corroborate the diagnosis, immunohistochemical staining was conducted, yielding the following outcomes: SMA (+++), h-caldesmon (+ focally), β-catenin (-), Ki-67 (20%), Desmin (-), and CD34 (-) (Fig. 2A∼2F). Consequently, the ultimate diagnosis aligns with a benign myopericytoma originating from the mesenchyme in the upper lip.

## Discussion

The term myopericytoma (MPC) was first introduced by Granter et al. in 1998 [4]. This category encompasses tumors in adults displaying features akin to infantile myofibromatosis, as well as glomangiopericytoma (GPC), glomus tumors, and angioleiomyoma (ALM). In the same year, Kutzner, from the Friedrichshafen Institute of Dermatopathology, introduced the concept of perivascular myoma, essentially referring to the same tumor [5]. In both instances, the tumor cells exhibit characteristics associated with perivascular myoid cells or myopericytic differentiation. MPC most commonly originates in the subcutaneous tissues of the skin, with a predilection for the distal extremities [2]. The lower limbs are the primary sites of occurrence, followed by the upper limbs, head, neck, and trunk [6–11]. In literature reports related to oral myopericytomas, there have been 6 cases occurring in the tongue, 10 cases in the salivary glands [3, 4, 6, 12–17], and 5 cases in the lips [7, 18–21]. There have been reports of a few cases occurring in AIDS patients with concurrent EBV infection [6]. Unlike those occurring in soft tissues, such as the limbs, EBV-positive myopericytomas tend to be located in specific regions, such as around the bronchi, tongue, and periprostatic area, and they may exhibit multifocality [22]. While most MPC cases are benign, there have been a few reported instances of malignancy or recurrence. Oral presentations are exceedingly rare [6, 10, 23, 24]. Typically, they manifest as solitary, well-defined, slow-growing, painless nodules [5, 25, 26]. However, multiple lesions can also occur [2]. Benign tumors typically measure less than 2 cm in diameter, although tumors that have recurred may reach dimensions of 5.6*5.5*3 cm [4]. To date, 41 cases of oral MPC have been reported, with ages of onset ranging from 21 months to 80 years and a median age of 43 years. The incidence rate ratio between males and females is 0.7:1. Furthermore, MPCs can occur multifocally across various anatomical regions [27]. Multiple-nodular tumors or deep-seated lesions generally demonstrate a greater degree of invasiveness in comparison to superficial nodules [4]. Malignant transformation within the oral cavity has been reported twice in the literature. In detailed, Terada reported a case of malignant tumor occurring in the buccal mucosa of a 61-year-old male. Through immunohistochemical and HE staining tests, a pathological diagnosis of low-grade malignant myopericytoma was made due to the presence of some atypical and scattered mitotic figures, positive p53 expression in tumor cells, and a relatively high Ki67 labeling index [28]. A malignant myopericytoma was reported to occur in the left neck region of an 80-year-old female, as documented by McMenamin. Intraoperatively, a tumor of approximately 20 mm was identified, invading into the sternocleidomastoid muscle. Nine months after excision, a tumor with morphological similarities measuring 70 mm was discovered in the liver, suggesting a metastatic lesion [29]. Ultrastructurally, tumor cells display characteristics consistent with pericytic differentiation [30]. In molecular genetics research, Dahle´n identified a t(7;12)(p21-22;q13-15) translocation in 5 cases of perivascular tumors. This translocation leads to the fusion of the ACTB gene on 7p22 with the GLI gene on 12q13 [31]. However, there have been no reports of extensive case series analyzing these lesions or providing a more comprehensive characterization of their biological potential. Some researchers, including Mito and Sadow, have proposed that BRAF mutations may constitute a novel genetic anomaly in the pathogenesis of myopericytomas, along with related biomarkers. Nevertheless, this viewpoint remains a topic of debate [32, 33]. 

A definitive diagnosis necessitates an excisional biopsy followed by histological examination. The primary characteristic of MPC is the concentric arrangement of pericytic myoid cells around thin-walled vascular channels [1]. In this case, H&E and alcian blue staining results reveal identical pathological features: relatively uniform oval or spindle-shaped myoid cells arranged in concentric circles or whirlpool patterns. Even in specific areas, mucinous degeneration is detected within the matrix between concentric circles or whirlpool structures. Additionally, some blood vessels contain occasional red blood cells, and certain tumor cells exhibit vacuolar changes with centrally located nuclei, which is typical feature of myopericytoma. Equally important is the presence of plump myoid cells encircling rounded, thin-walled vessels. In the current case, the site of onset is on the upper lip. Therefore, clinical differential diagnoses include fibrous hyperplasia, fibrous histiocytoma, pyogenic granuloma, minor salivary gland diseases (such as mucous cysts, adenomatoid hyperplasia), neurofibroma, lymphangioma, etc. Fibrous histiocytoma also presents as solitary, painless, well-defined nodular masses [34]. Pyogenic granuloma typically manifests as exophytic lesions with smooth or lobulated surfaces, presenting as pedunculated or sometimes sessile small red nodules on a base, often with a tendency to bleed [35]. Mucocoeles commonly occur on the lower lip, located beneath the mucosa, with a surface covered by a thin layer of mucosa, often appearing as translucent vesicles resembling water blisters, with a soft and elastic texture [36]. Adenomatoid hyperplasia is a rare non-neoplastic proliferation of minor salivary glands, presenting as asymptomatic, firm, papillary, non-tender nodular masses without ulceration [37]. Approximately 6.5% of neurofibromas involve oral lesions, with the tongue being the most commonly affected site and a low incidence rate in the lips. They appear as soft, flesh-colored nodules with characteristic central umbilication [38]. Lymphangiomas are benign developmental malformations characterized by abnormal proliferation of lymphatic vessels, relatively uncommon in oral lesions, with the lips being a rare site of involvement [39].While clinical features may appear similar, microscopic features are distinctive. Fibrous proliferation is characterized by mature connective tissue stroma [26]. The H&E-stained sections of fibrous histiocytoma reveal stratified squamous epithelium along with connective tissue stroma. The connective tissue stroma exhibits a dual cell population of fibroblasts and histiocytes. Fibroblasts are spindle-shaped and arranged in a twisted pattern (intersecting fibroblastic cell clusters) [34]. Pyogenic granuloma is identified by numerous dilated capillaries within a loose inflammatory stroma [35]. In H&E-stained sections of Mucocoeles, granulation tissue surrounding the mucoid cystic cavity is commonly observed, along with diffuse extrusion of mucin into the interstitial spaces [36]. Histopathological findings of adenomatoid hyperplasia include enlarged mucous gland acini, filled with secretory granules. Cell nuclei are compressed at the basal portion, accompanied by focal inflammation and ductal dilation [37]. Neurofibroma histology reveals an encapsulated lesion composed of proliferating neural elements with a background of mucin and enlarged cells. Lymphangiectasia is characteristic of lymphangioma [38]. Additionally, odontogenic lesions can be ruled out in lip lesions since they lack odontogenic epithelium [26]. Similarly, giant cell lesions are typically ruled out; however, there are rare reports of osteoclast-like giant cell tumors occurring in the lips [38].

Histological differential diagnoses include conditions such as myofibroma, glomus tumor, angioleiomyoma, and solitary fibrous tumor, especially myofibroma [4]. Glomus tumors are composed of cuboidal cells with clear cell borders and round central nuclei, lacking spindled cell morphology [5]. Angioleiomyomas show significant proliferation of thick-walled vessels [26]. Histologically, myofibromas exhibit nodular or multinodular growth patterns and distinct zonation: consisting of a peripheral area with lightly stained cells and a central area with darker staining, the proportions of which vary within the tumor. The peripheral area comprises spindle cells arranged in nodular or short fascicular patterns, with eosinophilic cytoplasm, morphologically intermediate between fibroblasts and smooth muscle cells. The central area is composed of round or polygonal primitive mesenchymal cells, arranged in solid sheets or around branching blood vessels resembling pericytes, with visible mitotic figures and necrosis [40–42]. Nevertheless, immunohistochemical differentiation is still necessary. In terms of immunohistochemistry, MPCs consistently demonstrate strong immunoreactivity for SMA, smooth muscle actin heavy chain, muscle-specific actin, and muscle-specific actin [5, 7, 43–45]. Moreover, the application of h-caldesmon in the diagnosis of leiomyoma is also quite important [46]. Most cases are negative for Desmin, although some occasional cases with focal Desmin reactivity have been reported. This suggests that possible precursor cell sources may include pericytic or myofibroblastic cells [5]. Strong SMA expression and negative CD34 staining effectively differentiate MPCs from other perivascular myoid tumors, such as solitary fibrous tumors and myofibromas [3, 21, 47–49]. The distinctive feature distinguishing MPC from angioleiomyoma is the concentric arrangement of cells and the absence of Desmin staining, a characteristic more frequently observed in MPC. As anticipated, we performed immunohistochemical testing on the specimen, including SMA, Desmin, and CD34. The test results are consistent with the reported characteristics of MPC in the literature: SMA (+++), Desmin (-), CD34 (-). It is noteworthy that a study by Matsuyama et al. demonstrated that up to 20% of angioleiomyoma cases exhibit focal perivascular concentric growth. This suggests that this feature alone cannot serve as a reliable means of differentiation between these two tumors. Additionally, while angioleiomyomas generally exhibit positive staining, they do not display positivity for Desmin within concentric cell structures. This necessitates further immunohistochemistry and consideration of molecular differences for improved discrimination between MPC and angioleiomyoma [50]. Differentiating MPC from epithelioid smooth muscle cell tumors, such as Pecomas, can be accomplished by observing their negativity for HMB45 [28]. 

Thus, pathological examination remains the gold standard for distinguishing MPC. Jo and colleagues conducted immunohistochemical staining for nuclear β-catenin in 50 soft tissue tumors located in the nasal sinuses or oral cavity. They recorded the staining intensity and extent in a semi-quantitative manner. Solitary fibrous tumors (SFTs) all exhibited nuclear β-catenin expression, and 90% of synovial sarcomas showed varying degrees of staining intensity from weak to strong. In contrast, none of the myopericytoma cases exhibited this expression [43]. Therefore, the immunohistochemical expression of nuclear β-catenin can be considered significant for distinguishing it from other diseases. In this case, the immunohistochemical staining results for nuclear β-catenin were also negative (Figure 2D), thereby excluding the possibility of solitary fibrous tumor. Immunohistochemical antibodies may lack absolute specificity, but they provide a degree of relative specificity. Multiple antibody combinations are frequently employed to enhance diagnostic accuracy. In the future, distinguishing these types of lesions may be facilitated through cytogenetics and/or molecular genetic studies. The immunohistochemical findings from the 41 cases are summarized in Table 1.

The standard treatment for benign MPC is surgical excision, typically resulting in a favorable prognosis [25, 51–54]. Extensive local excision is recommended to prevent recurrence, followed by meticulous follow-up. Among the 41 reported cases, only two patients experienced recurrences [4, 7]. These recurrences typically stem from unclear margins, tumor extension into other areas, or exceedingly rare malignant transformations. A single case report detailed an MPC situated in the parotid area, which recurred as a larger and multicentric lesion after two incomplete excisions [4]. While benign MPCs occurring in the lips are relatively straightforward to surgically remove, their removal often necessitates considering the patient’s age, lesion location, and potential postoperative adverse reactions. Compared to the case reported by Porat Ben Amy, which involved a 6-year-old boy with an MPC in the upper jaw involving teeth, our patient—a 7-year-old girl with a lesion in the upper lip that did not involve teeth—underwent a relatively straightforward excision [25]. Given the child’s young age, we chose a conservative direct excision, and she exhibited an excellent recovery during a three-month follow-up visit, showing no signs of recurrence.

### Electronic supplementary material

Below is the link to the electronic supplementary material.

Supplementary Material 1

Supplementary Material 2

Supplementary Material 3

## Data Availability

Data is provided within the manuscript or supplementary information files.

## Abbreviations

MPC: Myopericytoma
GPC: Glomangiopericytoma
ALM: Angioleiomyoma
NA: Not available
H&E: Hematoxylin and Eosin
