# Supplementary figures and images for: Pediatric upper lip myopericytoma: a case report and comprehensive review
Source: BMC Oral Health. 2024 Apr 20;24:478. doi: 10.1186/s12903-024-04106-y (PMC11031849; doi:10.1186/s12903-024-04106-y)

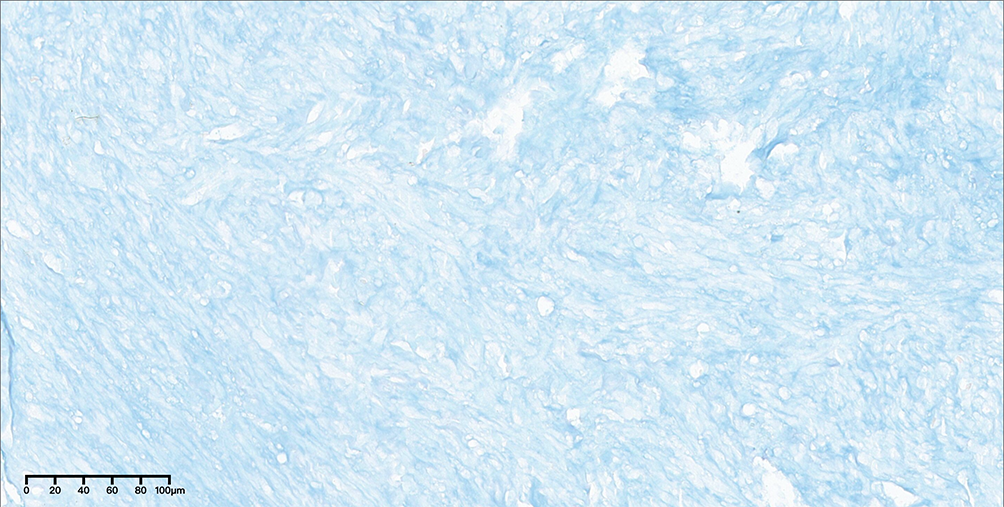

Supplement: Supplementary file 1 — Supplementary Material 1 [file 12903_2024_4106_MOESM1_ESM.png]
