# Supplementary material for: Pediatric upper lip myopericytoma: a case report and comprehensive review
Source: BMC Oral Health. 2024 Apr 20;24:478. doi: 10.1186/s12903-024-04106-y (PMC11031849; doi:10.1186/s12903-024-04106-y)
Supplement: Supplementary file 2 — Supplementary Material 2 [file 12903_2024_4106_MOESM2_ESM.docx]

Supplementary Table 1

The demographic features of myopericytoma reported in the oral cavity

| Category | Cases/Mean | Percentage |
| --- | --- | --- |
| Gender |  |  |
| Male | 17 | 41.46% |
| Female | 24 | 58.54% |
| Ages |  |  |
| < 18 | 6 | 14.63% |
| 18~60 | 25 | 60.98% |
| >60 | 10 | 24.39% |
| The average age | 42.2 |  |
| The site of onset |  |  |
| Parotid | 10 | 24.38% |
| Lip | 7 | 17.07% |
| Neck | 6 | 14.63% |
| Tongue | 5 | 12.20% |
| Buccal | 4 | 9.76% |
| Mandible/Submandibular | 3 | 7.32% |
| Temporal region | 3 | 7.32% |
| Other | 3 | 7.32% |

a. **Cases classified by gender, ages, and anatomical site are summarized.**
