# Supplementary material for: Pediatric upper lip myopericytoma: a case report and comprehensive review
Source: BMC Oral Health. 2024 Apr 20;24:478. doi: 10.1186/s12903-024-04106-y (PMC11031849; doi:10.1186/s12903-024-04106-y)
Supplement: Supplementary file 3 — Supplementary Material 3 [file 12903_2024_4106_MOESM3_ESM.docx]

**Supplementary figure 1 legend**

**【Supplementary figure 1 legend】mucinous degeneration was detected within the matrix between whirlpool structures by alcian blue staining (bar=100μm).**
